# The Transient Pubertal Free Thyroxine Dip Should Not Be Misinterpreted as Central Hypothyroidism: A Case Series

**DOI:** 10.1159/hrp/adaag031

**Published:** 2026-10-01

**Authors:** Mark R. Garrelfs, Jacquelien J. Hillebrand, Annemieke C. Heijboer, Christiaan F. Mooij, A.S. Paul van Trotsenburg, Nitash Zwaveling-Soonawala

**Affiliations:** a Department of Pediatric Endocrinology, Emma Children’s Hospital, Amsterdam University Medical Centers, University of Amsterdam and Vrije Universiteit, Amsterdam, The Netherlands; b Amsterdam Gastroenterology, Endocrinology and Metabolism, Amsterdam, The Netherlands; c Department of Laboratory Medicine, Endocrine Laboratory, Amsterdam University Medical Centers, University of Amsterdam and Vrije Universiteit Amsterdam, Amsterdam, The Netherlands; d Amsterdam Reproduction and Development Research Institute, Amsterdam, The Netherlands

**Keywords:** Central hypothyroidism, Puberty, Free thyroxine, Pituitary, Thyroid

## Abstract

**Introduction:**

During puberty, free thyroxine (fT4) concentrations transiently decrease with a nadir around Tanner stage 3. Due to the interindividual variation in timing of pubertal onset, this transient fT4 dip occurs at different ages and becomes less apparent within an age-specific reference interval (RI). This may result in fT4 concentrations temporarily falling below the lower limit of the RI when age-specific instead of Tanner stage-specific RIs are used. As thyroid stimulating hormone (TSH) levels usually remain within the age-specific RI, the resulting biochemical profile resembles central hypothyroidism and frequently triggers extensive, but often unnecessary diagnostic work-ups.

**Case Presentation:**

We present two cases of asymptomatic adolescents with low fT4 concentrations in combination with a normal TSH, in whom central hypothyroidism was initially suspected but subsequently excluded. In all cases, fT4 concentrations normalized later in puberty without intervention.

**Conclusion:**

This phenomenon likely reflects increased peripheral conversion of T4 to tri-iodothyronine (T3) under the influence of growth hormone and sex steroids. Awareness of this physiological phenomenon is essential to avoid misdiagnosis, overtreatment, and undue psychological stress. Our findings highlight the need for Tanner stage-specific fT4 RIs to guide thyroid function interpretation in adolescents.

**Established Facts**

Serum free thyroxine (fT4) concentrations reach a temporary physiological nadir during puberty (Tanner stage 3).

**Novel Insights**

This transient pubertal fT4 dip is not accurately reflected in age-specific RIs, causing serum fT4 levels to occasionally fall below the lower limit of the age-specific RI during puberty.

As serum thyroid stimulating hormone concentrations generally remain within age-specific reference intervals (RI) during puberty, the transient pubertal fT4 dip biochemically resembles central hypothyroidism.

Considering the transient pubertal fT4 dip in the differential diagnosis of asymptomatic adolescents with decreased plasma fT4 concentrations is crucial to prevent unnecessary diagnostic procedures and treatments.

## Introduction

The hypothalamus-pituitary-thyroid (HPT)-axis undergoes several age-related changes during childhood. In the neonatal period, serum free thyroxine (fT4) rises after the thyroid stimulating hormone (TSH) surge and peaks around day 3 of life followed by a gradual decline over several weeks [1]. Multiple studies have shown that in the years thereafter, fT4 levels slightly decline until adulthood [2–4].

A lesser known phenomenon is the transient decrease in fT4 concentrations during puberty, which is most pronounced around Tanner stage 3 [5–7]. This fT4 “dip” tends to be obscured in age-specific reference intervals (RIs) because the interindividual variation in timing of pubertal onset causes the effect of puberty on fT4 levels to average out across an age group [8]. The wider the age-range of a RI, the less apparent the effect of puberty on fT4 levels becomes. When age-specific (instead of Tanner stage-specific) RIs are used, this may result in fT4 concentrations temporarily falling below the lower reference limit [5]. Figure 1 conceptually illustrates how the physiological pubertal fT4 dip may lead to different interpretations of thyroid function test results when age-specific rather than Tanner stage-specific reference intervals are used. Because the displayed reference intervals are derived from separate studies, the figure should be interpreted as a schematic representation rather than a direct analytical comparison.

**Fig. 1.**
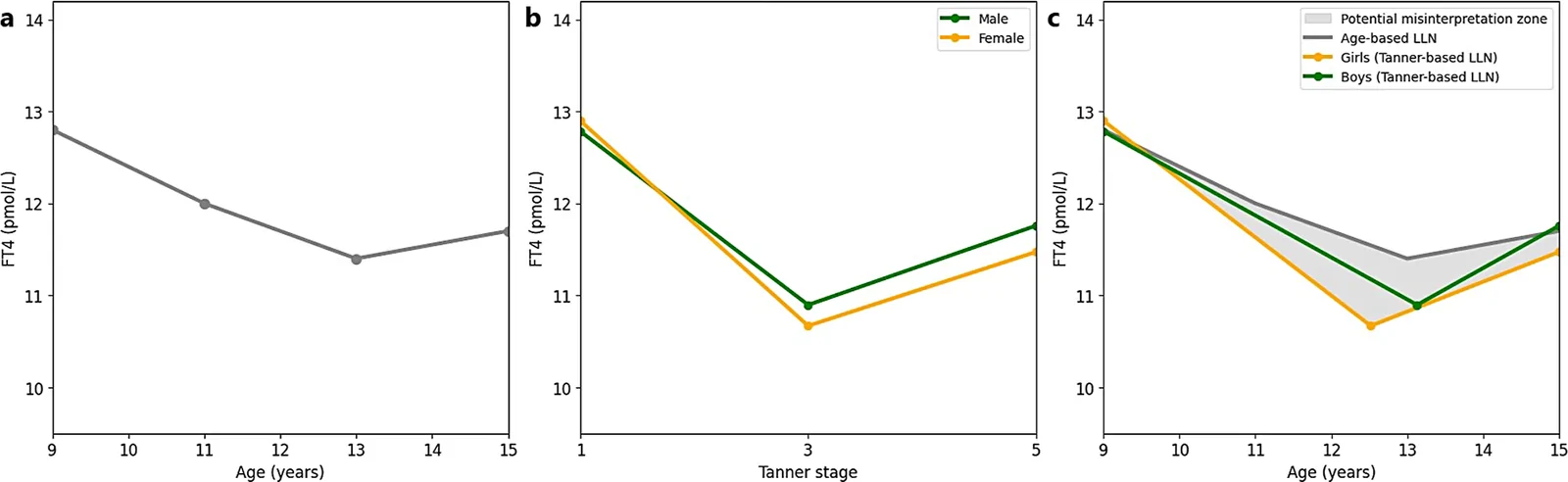
Conceptual illustration of differences between age-specific and Tanner stage-specific lower reference limits for serum free thyroxine (fT4). **a** Age-specific fT4 LLN (Cobas, Roche), for boys and girls combined, based on data derived from Jansen et al. [8]. **b** Tanner stage-specific fT4 LLN (Cobas, Roche), calculated as mean − 1.96 SD based on data derived from Surup et al. [5]. **c** Schematic comparison of age-specific (grey) and Tanner stage-specific lower reference limits for girls (orange) and boys (green). Age at Tanner stage 3 was based on the mean age reported by Surup et al. [5]. The shaded area represents the region in which fT4 concentrations may fall below the age-specific lower reference limit while remaining within the Tanner stage-specific reference interval. The figure is intended to illustrate the potential impact of reference interval selection on interpretation of thyroid function test results during puberty. Because the displayed reference limits are derived from separate published studies, the figure should be interpreted as a conceptual representation rather than a direct analytical comparison.

A low fT4 concentration in combination with a non-elevated TSH can be a cause of concern as it biochemically mimics central hypothyroidism [9]. When central hypothyroidism is suspected, an elaborate diagnostic work-up is usually performed to rule out (acquired) hypothalamic or pituitary conditions [10, 11]. However, in many cases, the observed thyroid function profile reflects normal physiology rather than a sign of disease. Knowledge and understanding of the changes in thyroid function during puberty can prevent costly over-diagnostics and unnecessary psychological stress.

To overcome this diagnostic challenge, Tanner stage–specific fT4 RIs should be used during puberty. Unfortunately, such RIs are currently not routinely available, forcing clinicians to interpret thyroid function test results in adolescents by using age-specific RIs that do not adequately reflect physiology. Using two illustrative cases, reported in line with the CARE guidelines (online suppl. file 1; for all online suppl. material, see https://doi.org/10.1159/hrp/adaag031), we aim to raise awareness of the transient pubertal fT4 dip and provide practical guidance for the interpretation and management of abnormal thyroid function test results during adolescence.

## Case Series

A 14-year-old boy with a history of attention deficit hyperactivity disorder was treated with dexamphetamine (12.5 mg/day). To rule out an organic cause of his attention deficit hyperactivity disorder, his psychiatrist screened for thyroid dysfunction. His fT4 was 9.8 pmol/L and TSH 1.8 mU/L (fT4 RI 11.7–20.3 pmol/L and TSH RI 0.9–5.1 mU/L, Cobas Roche Diagnostics) (Fig. 2). At that time, free tri-iodothyronine (fT3) level was 4.1 pmol/L (RI 2.8–5.3 pmol/L, Alinity, Abbott Diagnostics), total T4 level was 53 nmol/L (Total T4 RI 65–140 nmol/L, Architect, Abbott Diagnostics) and thyroid binding globulin (TBG) level was 210 nmol/L (TBG RI 200–650 nmol/L, RIA, Thermo Fisher Scientific Inc.). He was referred to our tertiary department of pediatric endocrinology for evaluation of suspected central hypothyroidism. There were no signs or symptoms suggestive of this diagnosis or other pituitary hormone deficiencies. With respect to puberty, he was in Tanner stage G3 with a bilateral testicular volume of 8 mL. To rule out a possible link with psychotropic drug use, dexamphetamine was stopped and 3 months later fT4 and TSH measurement was repeated. No clear difference was observed (fT4 11.1 pmol/L; TSH 2.1 mU/L). Psychotropic drugs were not restarted. Additional endocrine diagnostics including low-dose ACTH testing (1 microgram), showed no evidence of other pituitary hormone deficiencies. MRI of the hypothalamus-pituitary region showed no abnormalities. Genetic testing ruled out variations in the five genes responsible for isolated congenital central hypothyroidism (*TBL1X*, *IGSF1*, *IRS4*, *TSHB*, *TRH-R*). The boy was treated with levothyroxine (LT4) from age 16 to 17. He did not notice any benefits of treatment and his TSH levels during treatment were never suppressed. Before his 18th birthday, LT4 was stopped and fT4 and TSH remained normal during follow-up (fT4 13.2 pmol/L, TSH 1.3 mU/L) (Fig. 2).

**Fig. 2.**
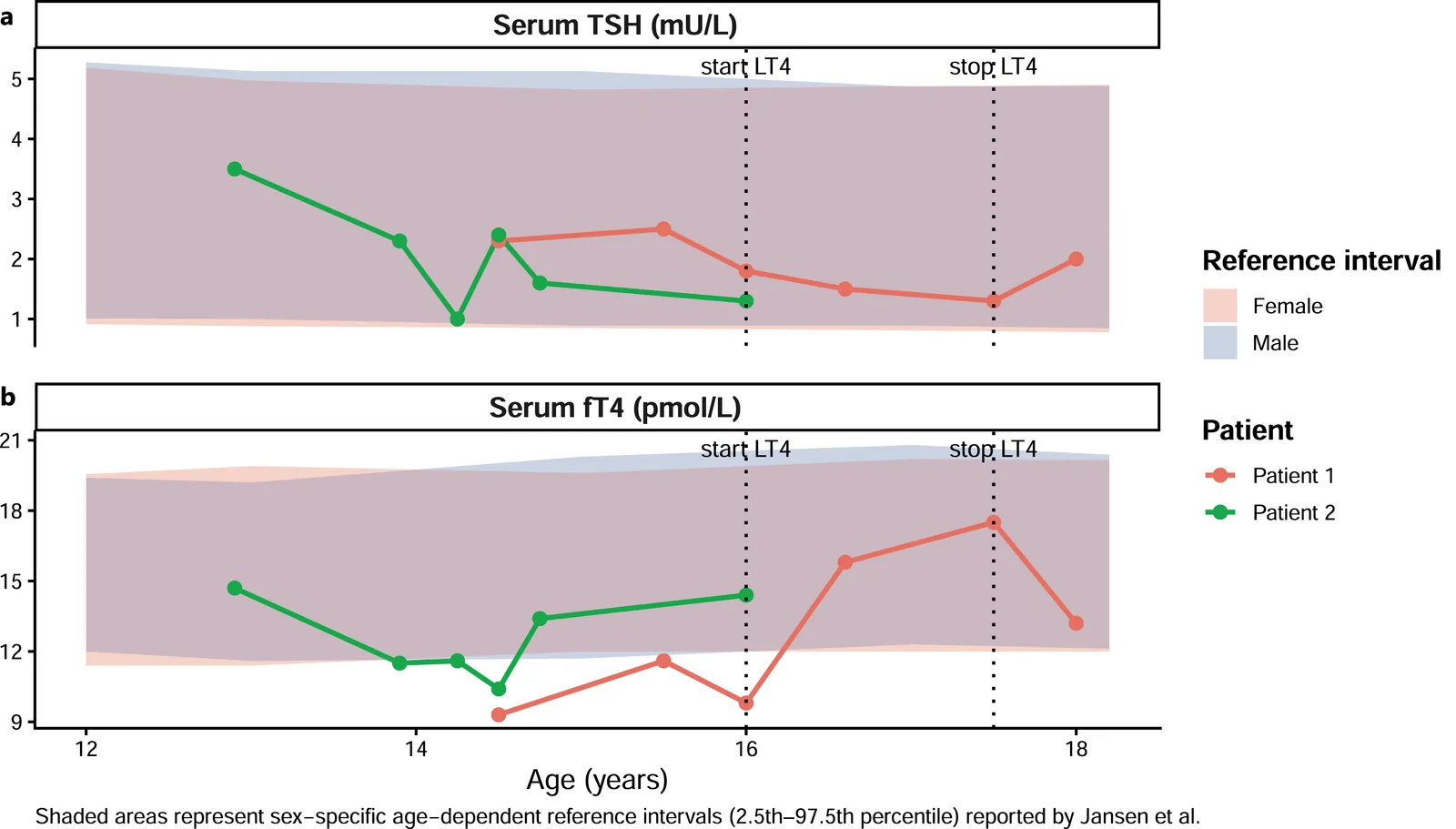
Follow-up of fT4 and TSH concentrations in two adolescents with transient hypothyroxinemia of puberty. **a** Serum TSH concentrations (mU/L) and (**b**) serum fT4 concentrations (pmol/L) over time in two adolescents. Shaded areas indicate the age- and sex-specific reference intervals (2.5th–97.5th percentile) as reported by Jansen et al. [8] Vertical dashed lines denote the start (16 years) and discontinuation (17.5 years) of levothyroxine (LT4) treatment in patient 1. fT4, free thyroxine; LT4, levothyroxine; TSH, thyroid stimulating hormone.

An 11-year-old girl was evaluated for proportionate short stature (height −2.5 standard deviation [SD] for age). Endocrine testing revealed no abnormalities. At that time, her thyroid function test results were normal (fT4 13.9 pmol/L; RI 11.4–19.2 pmol/L, TSH of 2.6 mU/L; RI 1.0–5.4 mU/L, Cobas, Roche Diagnostics). To test for Turner syndrome, fluorescence in situ hybridization was performed on 205 peripheral blood interphase nuclei using probes targeting the CEP X/CEP Y (Vysis-Abbott) loci. The fluorescence in situ hybridization result (nuc ish Xp11.1q11.1(DXZ1x2)[194]/(DXZ1x1)[11]) revealed low grade (11/205 ≈ 5%) 45,X/46,XX mosaicism. Because of the diagnosis short stature due to Turner syndrome, she was started on growth hormone treatment 1.4 mg/m2/day. Growth hormone treatment was tolerated well and her thyroid function test results remained normal during treatment. Biochemically, there were no signs of hypergonadotropic hypogonadism and puberty started spontaneously at the age of 12 years. Thereafter, puberty progressed normally and menarche followed at the age of 13 years and 3 months with a regular menstrual cycle soon thereafter. Periodic screening according to Turner syndrome guidelines revealed a decline in serum fT4 during puberty with a nadir of 10.4 pmol/L with a TSH level of 2.4 mU/L (Fig. 2) at Tanner stage M3. At that time, her serum fT3 concentration was 5.8 pmol/L (RI 2.8–5.3 pmol/L, Alinity, Abbott Diagnostics), serum total T4 concentration was 84 nmol/L (Total T4 RI 65–140 nmol/L, Architect, Abbott Diagnostics) and TBG concentration was 450 nmol/L (TBG RI 200–650 nmol/L RIA, Thermo Fisher Scientific Inc.). TSH levels remained within the RI. The girl had no signs or symptoms of central hypothyroidism, and biochemically there was no evidence of other pituitary hormone deficiencies. At the age of 14 years and 9 months, while still being treated with growth hormone, fT4 normalized (13.4 pmol/L and TSH 1.6 mU/L). Her height increased from −2.5 SD to −0.7 SD during treatment and growth hormone was stopped at the age of 15 years. Thyroid hormone levels remained within the age-specific RI during follow-up (Fig. 2).

## Discussion

Here we present two cases of asymptomatic children with a transient serum fT4 dip during puberty. Their thyroid function profile resembled central hypothyroidism and was the reason for an elaborate diagnostic work-up. No abnormalities were found and fT4 concentrations returned to levels within the age-specific RI near the end of puberty. We, therefore, concluded that the transient low fT4 concentrations represented a physiological phenomenon.

Serum fT4 concentrations depend on several factors including (but not limited to) age, sex, Tanner stage, body mass index, and ethnicity [2, 3, 5, 8]. Numerous studies on pediatric thyroid hormone RIs have been performed, but the results are difficult to compare due to differences in study population, study design, age partitioning and assays used. Nevertheless, most studies show a similar pattern characterized by decreasing serum fT4 concentrations with increasing age, reaching a (temporary) nadir at puberty [5–8, 12–28]. The decrease in fT4 levels is accompanied by a rise in serum (free) T3, which seems to be more pronounced in boys than in girls [4, 5, 7, 12, 16, 18, 19, 25].

Only a few studies on pediatric RIs have related thyroid hormone concentrations to Tanner stage. Dunger et al. performed the only longitudinal study till date and measured fT4 in serum from 39 healthy children between the ages of 10 and 15 years with 6-monthly intervals [6]. They found a decrease of fT4 from 15.7 ± 0.6 pmol/L at age 10 years to 13.0 ± 0.6 (*p* < 0.001) at age 12.5 years, before rising to 15.9 ± 0.7 at age 15 years. In boys, fT4 decreased from 16.3 ± 0.6 pmol/L at age 10 years to 14.3 ± 0.3 at age 13.5 years, and rose again to 15.4 ± 0.5 at age 15 years. In both sexes, the nadir occurred at Tanner stages 3–4. Marwaha et al. performed a cross-sectional study evaluating thyroid function in 2134 children free of thyroid disease and found that serum fT4 levels decreased with the onset of puberty in both boys and girls and remained stable after Tanner stage 3 [7]. Surup et al. generated age- and sex-specific pediatric reference intervals for TSH, fT3, and fT4 using samples from 3140 participants (age range: 3 months−18 years). The authors showed strong age and sex effects and clear modulation by puberty: TSH generally declines with age and across puberty; fT3 falls with age but shows a temporary peak around Tanner stage 3 in boys; and fT4 shows a pronounced dip at Tanner stage 3 in both sexes before rising again toward late puberty. Although the authors did not explicitly provide the 2.5th and 97.5th percentiles of fT4 by Tanner stage, they can be calculated from the given mean and SD by assuming a normal distribution (which is generally true for fT4). For males (14.64 ± 1.91 pmol/L) and females (14.53 ± 1.97 pmol/L) in Tanner stage 3, this yields intervals of 10.9–18.4 pmol/L and 10.7–18.4 pmol/L, respectively (using the Cobas assay from Roche Diagnostics, module 401 and 601). The upper and lower limits of the Tanner stage-specific RIs are considerably lower than those of the corresponding age-specific RIs. Using age-specific RIs instead of Tanner-stage specific RIs can, therefore, lead to misinterpretation of thyroid function test results (Fig. 1).

Of note, a limitation of the comparison between age-specific and Tanner-stage specific RIs as presented in Figure 1, is that the data were derived from different studies. Although both studies used Roche Cobas fT4 platforms, inter-and intra-assay variability due to differences in, for example, analyzer generation, calibration procedures and reagents used cannot be excluded. Furthermore, RIs were established using the direct method in the study by Surup et al. while the indirect method was used in the study by Jansen et al. Therefore, the figure should be interpreted as a conceptual illustration of the discrepancy between age-based and pubertal-stage-based reference intervals rather than a direct analytical comparison.

The exact etiology of this phenomenon remains to be elucidated, but may result from the effects of growth hormone and sex steroids on iodothyronine deiodinases. Sex steroids and growth hormone secretion both increase during puberty, with peak levels of IGF-1 coinciding with the fT4 nadir [29]. Growth hormone is known to upregulate iodothyronine deiodinase 2 (DIO2), which converts T4 to T3 in peripheral tissues and the pituitary [30]. In animal studies, sex steroids also seem to have a direct regulatory effect on DIO activity but to what extent this contributes to the observed changes in thyroid hormone levels in humans remains uncertain [31–33]. Increased peripheral conversion of T4 to T3 by upregulated DIO2 could theoretically lead to lower plasma T4 concentrations while maintaining adequate tissue T3 levels. As DIO2 is also present in the pituitary, negative T3 feedback on thyrotrope cells may prevent a significant TSH rise. These changes result in a temporary new HPT-axis balance during puberty. Indirect evidence for this theory is provided by studies on thyroid function changes in girls with precocious puberty and the subsequent effects of treatment with GnRH-analogs. In girls with precocious puberty, lower fT4 values are found and these changes are reversed after starting treatment with GnRH-analogs [34, 35]. The same pattern was described before and after starting GnRH-analogs to postpone puberty in children born small for gestational age that were treated with growth hormone [16]. It is uncertain to what extent treatment with growth hormone has influenced the pubertal fT4 concentrations of the patient presented in case 2. Theoretically, treatment with growth hormone could have exacerbated the proposed effects on the peripheral conversion of T4 to T3 by influencing DIO activity. In our clinical experience, children treated with growth hormone do not seem to present with fT4 levels below the age-appropriate RI during puberty more often than children that are not on exogenous growth hormone. Nevertheless, physicians should consider the possible effects of growth hormone treatment on thyroid hormones when interpreting test results.

Interestingly, the absence of a compensatory rise in TSH despite lower circulating fT4 concentrations contrasts with the classical log-linear relationship between fT4 and TSH described in primary hypothyroidism [36–38]. In individuals with an intact hypothalamic-pituitary-thyroid axis, relatively small decreases in circulating fT4 typically result in substantial increases in TSH secretion. During the pubertal fT4 dip, however, TSH concentrations generally remain within the reference interval. This observation supports the hypothesis that intracellular thyroid hormone availability (within the hypothalamus and pituitary) remains adequate, possibly because of increased local conversion of T4 to T3 through enhanced DIO activity.

Because the fT4 RI is wide, only a small proportion of children develop fT4 levels below the age-appropriate RI. Individuals with a naturally lower HPT-axis set-point, with fT4 levels near the lower limit of the established RI, are more likely to dip below this limit during puberty [39]. Perhaps, other factors like deiodinase polymorphisms that influence sensitivity to the effects of sex steroids and growth hormone may also play a role. It is important to keep in mind that reference intervals are statistical constructs describing the distribution of results within a reference population and do not define biological normality. By definition, approximately 2.5% of healthy individuals will have fT4 values below the lower limit of a 95% reference interval. This distinction is particularly relevant during puberty, when substantial interindividual variation in developmental timing further broadens the physiological distribution of fT4 concentrations. When low fT4 concentrations are encountered in an otherwise healthy pubertal child, the diagnostic approach should be concise and focused on excluding “true” (i.e., pathologic) central hypothyroidism (Fig. 3). We advise confirming the presence of a persistently low fT4 concentration by performing serial measurements. When a persistently low fT4 concentration is confirmed an endocrine work-up consisting of fT4, TSH, IGF-1, prolactin and early morning LH, FSH, estradiol, or testosterone is advised. In addition, we advise to check for prior, preferably prepubertal, fT4 measurements to obtain information about the patients HPT-axis set-point. We do not recommend performing routine measurements of fT3, as fT3 levels do not adequately discriminate between physiology and central hypothyroidism [40, 41].

**Fig. 3.**
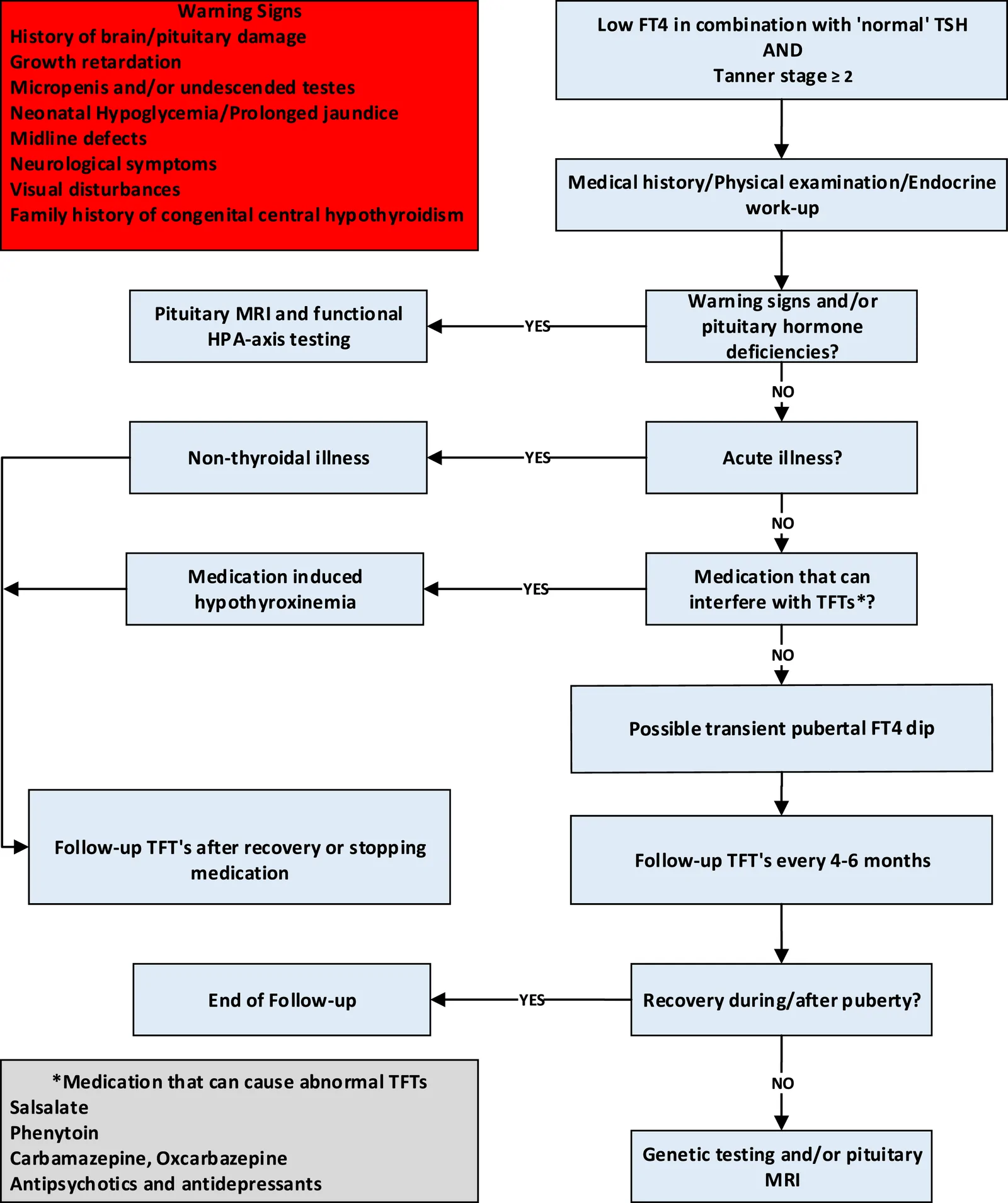
Suggested diagnostic work-up for pubertal patients with a biochemical profile resembling central hypothyroidism (when using age-specific reference intervals for interpretation of thyroid function test results). fT4, free thyroxine; LT4, levothyroxine; MRI, magnetic resonance imaging; TFT, thyroid function test; TSH, thyroid stimulating hormone. *List is not exhaustive [42, 43].

No definitive evidence-based fT4 cut-off can be established; however, in line with the findings of Surup et al., fT4 concentrations of less than 10.5 pmol/L (Cobas, Roche Diagnostics) may indicate pathology. Of note, both cases described in this paper showed transient (non-pathological) fT4 concentrations below 10.5 pmol/L. This could be explained by the fact that fT4 is normally distributed in the population which, by definition, implies that 2.5% of healthy subjects have fT4 concentrations below the lower limit of the 95% RI. In our clinical experience, LT4 treatment of children with a transient pubertal fT4 dip does not seem to be beneficial (most likely because it reflects a physiological phenomenon). In retrospect, initiation of LT4 therapy in case 1 was based primarily on biochemical findings rather than clinical symptoms, growth abnormalities, or evidence of pituitary dysfunction. This case thus illustrates the potential risk of over-medicalization. Nevertheless, in cases where there is doubt whether or not the persons abnormal TFTs represent disease or physiology, the effect of LT4 treatment on TSH levels can provide additional information. While in true central hypothyroidism, treatment with low-to normal levothyroxine doses (where fT4 is kept within the age-appropriate or preferable Tanner stage-specific RI) leads to TSH suppression, this would not be expected in case of a transient pubertal fT4 dip. In questionable cases where TSH stays relatively unchanged during treatment, stopping LT4 after puberty should be considered. Treatment should always be preceded by a formal evaluation of the hypothalamus-pituitary-adrenal axis to prevent an adrenal crisis in case of central hypothyroidism as part of multiple pituitary hormone deficiencies.

When no warning signs are present and the endocrine work-up shows no signs of pituitary insufficiency, the physiologic transient pubertal fT4 dip is the most likely cause of the biochemical profile. Follow-up of thyroid function, growth and the development of cause specific symptoms may subsequently be performed every 4–6 months. If symptoms arise or fT4 progressively declines, the diagnosis should be revisited and further testing be considered. This should also be the case when thyroid function fails to normalize after puberty.

Misinterpreting physiological changes for endocrinopathies can be avoided by using appropriate RIs. Although Tanner stage-specific reference intervals would provide the most physiologically accurate interpretation, implementation in routine laboratory practice remains challenging because Tanner stage information is rarely available to laboratory professionals. An approach to implementation could be the incorporation of interpretative comments on laboratory reports for pubertal-aged children and increasing awareness among clinicians. Development of robust Tanner stage-specific reference intervals remains an important long-term goal.

## Conclusion

Serum fT4 concentrations reach a physiological nadir during puberty (Tanner stage 3), which is not accurately reflected in age-specific RIs. This can cause serum fT4 levels to occasionally fall below the lower limit of the age-specific RI, especially when prepubertal fT4 levels were already close to the lower limit of the RI. Using Tanner stage specific RIs could solve this issue but, unfortunately, these are currently not widely available and practical implementation is challenging. Considering this transient pubertal fT4 dip in the differential diagnosis of asymptomatic adolescents with decreased plasma fT4 concentrations is crucial to prevent unnecessary diagnostic procedures and treatments.

## Statement of Ethics

This study was performed in accordance with the Declaration of Helsinki. Ethical approval was not received for this human study because Ethical approval is not required for this study in accordance with local or national guidelines. Written informed consent was obtained from participants, all above the age of 18 years at the time of consent, for publication of the details of their medical case.

## Conflict of Interest Statement

The authors have no conflicts of interest to declare.

## Funding Sources

This study was not supported by any sponsor or funder.

## Author Contributions

Mark R. Garrelfs performed data acquisition, designed the figures, and drafted the article. Jacquelien J. Hillebrand, Annemieke C. Heijboer, Christiaan F. Mooij, Nitash Zwaveling-Soonawala, and A.S. Paul van Trotsenburg reviewed and edited the manuscript.

## Data Availability

All data generated or analyzed during this study are included in this article and its online supplementary material. Further enquiries can be directed to the corresponding author.
